# Identification of diagnostic biomarkers in patients with gestational diabetes mellitus based on transcriptome gene expression and methylation correlation analysis

**DOI:** 10.1186/s12958-019-0556-x

**Published:** 2019-12-27

**Authors:** Enchun Li, Tengfei Luo, Yingjun Wang

**Affiliations:** 1 Department of Gynecologic Oncology, Women’s Hospital, School of Medicine, Zhejiang University, No. 1 Xueshi Road, Hangzhou, 310006 China; 2 Department of Obstetrics, Hangzhou Women’s Hospital, Hagzhuo, China; 3 Department of Obstetrics, Women’s Hospital, School of Medicine, Zhejiang University, Hangzhou, China

**Keywords:** GDM, Methylation, Pathway, Diagnostic markers, SVM

## Abstract

**Background:**

Gestational diabetes mellitus (GDM) has a high prevalence in the period of pregnancy. However, the lack of gold standards in current screening and diagnostic methods posed the biggest limitation. Regulation of gene expression caused by DNA methylation plays an important role in metabolic diseases. In this study, we aimed to screen GDM diagnostic markers, and establish a diagnostic model for predicting GDM.

**Methods:**

First, we acquired data of DNA methylation and gene expression in GDM samples (*N* = 41) and normal samples (N = 41) from the Gene Expression Omnibus (GEO) database. After pre-processing the data, linear models were used to identify differentially expressed genes (DEGs). Then we performed pathway enrichment analysis to extract relationships among genes from pathways, construct pathway networks, and further analyzed the relationship between gene expression and methylation of promoter regions. We screened for genes which are significantly negatively correlated with methylation and established mRNA-mRNA-CpGs network. The network topology was further analyzed to screen hub genes which were recognized as robust GDM biomarkers. Finally, the samples were randomly divided into training set (*N* = 28) and internal verification set (*N* = 27), and the support vector machine (SVM) ten-fold cross-validation method was used to establish a diagnostic classifier, which verified on internal and external data sets.

**Results:**

In this study, we identified 465 significant DEGs. Functional enrichment analysis revealed that these genes were associated with Type I diabetes mellitus and immunization. And we constructed an interactional network including 1091 genes by using the regulatory relationships of all 30 enriched pathways. 184 epigenetics regulated genes were screened by analyzing the relationship between gene expression and promoter regions’ methylation in the network. Moreover, the accuracy rate in the training data set was increased up to 96.3, and 82.1% in the internal validation set, and 97.3% in external validation data sets after establishing diagnostic classifiers which were performed by analyzing the gene expression profiles of obtained 10 hub genes from this network, combined with SVM.

**Conclusions:**

This study provided new features for the diagnosis of GDM and may contribute to the diagnosis and personalized treatment of GDM.

## Introduction

Gestational diabetes mellitus (GDM) is a common pregnancy complication associated with various perinatal conditions, including pre-eclampsia, cesarean section, macrosomia, birth injury, and neonatal hypoglycemia [1]. About 6 to 9% of pregnancies are associated with GDM [2], and the prevalence of undiagnosed Type II diabetes among women of childbearing age has increased due to the increased obesity and Type II diabetes in recent years [3]. Therefore, the International Association of Diabetes and Pregnancy Research Groups (IADPSG) recommended that women diagnosed with diabetes based on early pregnancy diagnostic criteria should be classified as epigenetics diabetic [1]. However, the criteria for GDM diagnosis are still controversial. Therefore it is essential to find an effective diagnostic method, and optimal medical and obstetric managements for reducing the adverse pregnancy outcomes of GDM.

DNA methylation is an epigenetic modification of cells, it can regulate gene expression without altering the gene sequence [4]. Although the relationship between gene expression and gene sequence is complex [5, 6], these methylation events can respond to nutritional and environmental effects, and modulate gene expression patterns based on the flexibility of epigenome modification [7, 8]. Thus, methylation can serve as potential biomarkers for early cell transformation [9]. In fact, it has been reported that serum DNA methylation can be considered as a biomarker for early detection of cancer, especially in the field of cancer. [10, 11]. Moreover, DNA methylation of specific genes (SEPT9, RASSF1A, APC, and GADD45a) has been proposed as a biomarker for the diagnosis and prognosis of colorectal cancer [12] and breast cancer [13].

The aim of this study was to integrate high-throughput methylation profiles and gene expression profiling data from a large number of patients to study altered DNA methylation patterns between GDM and healthy pregnant women. In addition, we aimed to identify specific DNA methylation sites as potential biomarkers and further establish a GDM diagnostic classifier.

## Materials and method

**Fig. 1:**
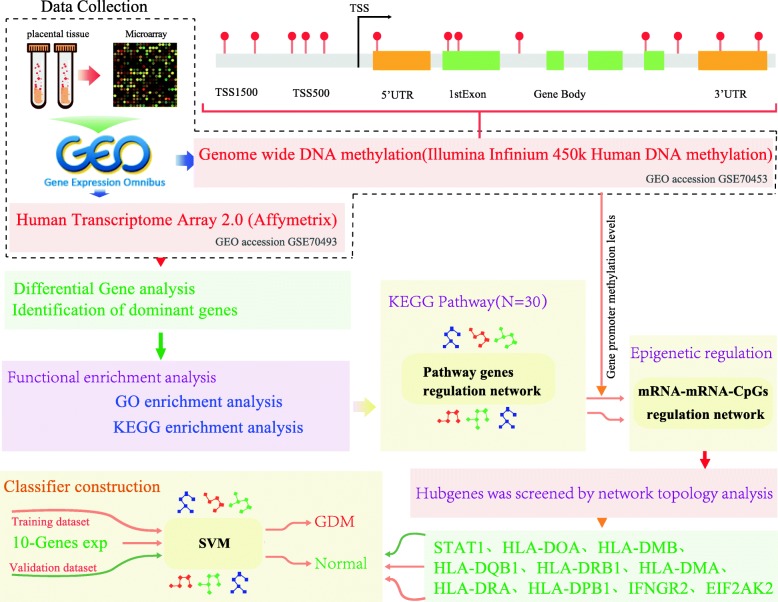
The workflow of the present study

In the present study, the analysis methods included the following steps: data collection, DEGs analysis, enrichment analysis, pathway interaction network, feature selection, and classifier construction and validation. The workflow was shown in Fig. 1.

### Data collection

Gene methylation and gene expression data were obtained from previous studies by Binder AM et al. [14], from the GEO database (http://www.ncbi.nlm.nih.gov/geo/). The gene methylation data was performed by the GPL13534 platform (Illumina HumanMethylation450 BeadChip), numbered as GSE70453. The data set contained a total of 82 samples, including 41 GDM samples and 41 normal placental tissues (Table 1). Samples were matched based on maternal age, pre-pregnancy BMI, method of conception, ethnicity, smoking status, and infant sex. Approximately 50% of these women were normal weight (18.5 ≤ BMI < 25) prior to pregnancy. Aside from two previously underweight mothers, the remaining women were either overweight (20%; 25 ≤ BMI < 30) or obese (29%; 30 ≤ BMI). Birth weight and gestational age were not associated with GDM in this study population. The gene expression profile data was acquired from GPL17586 platform (Affymetrix Human Transcriptome Array 2.0), numbered as GSE70493. The data set contained a total of 63 samples from the same batch of samples for detecting methylation data, of which 32 were GDM samples and 31 were healthy placental tissues.

**Table 1:** Characteristics of placenta samples were assessed on the Illumina Infinium Array

Characteristic	Cases (n = 41)	Controls (n = 41)	p-value
Pre-pregnancy BMI (kg/m^2^)	26.653 (5.733)	26.410 (5.422)	*p* = 0.7635
Maternal Age (years)	33.171 (4.652)	33.487 (4.853)	p = 0.7635
Gravidity			*p* = 0.3891
1	8 (19.51%)	4 (9.76%)	
2	11 (26.83%)	16 (39.02%)	
3	13 (31.71%)	10 (24.39%)	
> 4	8 (19.51%)	11 (26.83%)	
Smoke during Pregnancy			
No	38 (92.683%)	38 (92.683%)	*p* = 1
Yes	3 (7.317%)	3 (7.317%)	
Infant Sex			
Males	20 (48.78%)	21 (48.78%)	p = 1
Females	21 (51.22%)	20 (51.22%)	
Ethnicity			
Non-Hispanic White	23 (56.098%)	23 (56.098%)	*p* = 0.9437
Hispanic or Latino	5 (12.195%)	4 (9.756%)	
Asian/Pacific Islander	7 (17.073%)	6 (14.634%)	
Black/African-American	6 (14.634%)	8 (19.512%)	
Conception			
Spontaneous planned	24 (58.537%)	24 (58.537%)	p = 1
Spontaneous unplanned	12 (29.268%)	12 (29.268%)	
Ovulation-induction drug	2 (4.878%)	2 (4.878%)	
IVF	3 (7.317%)	3 (7.317%)	
Gestational Age (weeks)	39.077 (0.932)	39.206 (1.047)	*p* = 0.5576

### Methylation data processing

We first downloaded the methylation Beta values of the normalized CpG sites and further converted it to the methylation M value. A total of 82 samples of the GDM and healthy group samples were included in the study cohort, and we further removed the sites with missing values​greater than 20% of all samples, as well as samples with missing values greater than 20% in each CpG sites. Then we used the impute R package [15] for missing value completion. Probes which were known to bind sex chromosomes, cross-hybridize to multiple locations, or target a single-nucleotide polymorphism (SNP) were removed, based on previous annotation [16, 17]. As the treatment of Zhang et al. [18], we further removed the methylation site from the non-promoter region, thus 236,070 probes for DNA methylation analysis were obtained. All analysis was performed by using M values​to improve the statistical calculation of methylation [19], though Beta values were also included in the tables for biological interpretation.

### Gene expression data processing

For gene expression data, we first downloaded the raw data of microarray data, removed the duplicated samples, and finally screened 30 GDM placental tissues and 25 healthy placental tissues. The oligo R package [20] was used for data processing to obtain probe expression profiles and further the RMA [21] method for data standardization. We finally obtained the expression matrix of 55 samples of 70,523 gene probes, and probe annotation was performed by the R package hta20transcriptcluster.db to remove probes matched to multiple genes (https://bioconductor.org/packages/release/data/annotation/html/hta20transcriptcluster.db.html). Multiple probes matched to one were used the median values as the expression of this modified gene. The expression profiles of 23,313 genes were finally obtained.

### Screening of significant DEGs

The R package limma [22] was used to screen DEGs between GDM samples and normal samples. The *p*-value < 0.05 as the threshold.

### Functional enrichment analyses

Gene Ontology (GO) and Kyoto Encyclopedia of Genes and Genomes (KEGG) pathway enrichment analysis was performed by using the R package clusterProfiler [23] for DEGs. To identify over-represented GO terms in three categories (biological processes, molecular function and cellular component), and KEGG pathway, we used the R package DOSE [24] to visualize. For both analyses, a *p*-value <  0.05 was considered to denote statistical significance.

### Construction of KEGG pathway gene interaction network

An XML file of the enriched KEGG pathway was downloaded from the KEGG [25] website. We used the R package XML to extract the relation, entry, and group relationships in these XML files. Then the script was used to extract the interaction information of these genes. We constructed the KEGG pathway gene interaction network, and used Cytoscape [26] software to visualize, and analyze the topological properties of the network.

### Screening for key epigenetics diagnostic genes in GDM

First, we extracted the methylation data of the samples which matching the gene expression profile, and further extracted the CpG methylation sites of the gene promoter region in the pathway network. By calculating the correlation between the promoter methylation site and gene expression, we selected a significantly negative correlation with the threshold of *p*-value < 0.05. Thus, we obtained the methylation site corresponding to the epigenetics driven gene and its promoter region. Based on the network interaction information of these genes and the relationship with CpG, the gene-gene-CpG network was visualized by using Cytoscape. The Degree, Closeness, and Betweenness in the network was calculated by using the plug-in of cytohubba [27] and the intersection genes of top 10 Degree, Closeness, and Betweenness were selected as the final key genes.

### Construction of GDM diagnostic prediction model and validation

A diagnostic prediction model based on the SVM [28] classification was built to predict GDM and normal healthy samples by feature-based genes. The SVM was a supervised learning model in machine learning algorithms that it can analyze data and identify patterns. It can construct a hyperplane, which can be used for classification and regression in high or infinite dimensional space. Given a set of training samples, each tag belongs to two categories. One SVM training algorithm builds a model and assigns new instances to one class or another, making it a non-probabilistic binary linear classification. We randomly and uniformly divided all samples into training data sets and validation data sets. The model was constructed in the training data set, and the ten-fold cross-validation method was used to verify the classification ability of the model. The established model was then used to predict the samples in the validation data set. The predictive power of the model was estimated by using the area under the ROC curve (AUC) and the model’s predictive sensitivity and specificity for GDM were analyzed.

### External data sets validate the clinical validity of the model

A set of Agilent-039494 SurePrint G3 Human GE v2 8x60K Microarray chipset dataset GSE128381 [29] with 183 Placental tissue samples, including 6 GDM patients, 177 normal samples, was selected as a separate external validation dataset. The standardized data was downloaded, and the expression profile of characteristic genes was extracted and substituted into the model to predict the samples and compare with the clinically detected diseases to analyze the accuracy of prediction, as well as the cross-platform of the model was verified. Furthermore, a random sample of 50% of normal samples is extracted one thousand times, and the expression spectrum of the characteristic gene is extracted from the model, and the prediction of the model is observed to observe the prediction stability of the model. The differences between pre-pregnancy age and pre-pregnancy BMI between the GDM-predicted and normal samples were compared. At the same time, a set of chip dataset GSE128381 [30] of the NuGO array platform was included, and sample generation into the model to predict the GDM samples and compared with the GDM identified by the underwent a 100 g 3 h Oral Glucose Tolerance Test (OGTT) between the 24 -34th gw method.

## Results

### Identification of DEGs between GDM and healthy samples

**Fig. 2:**
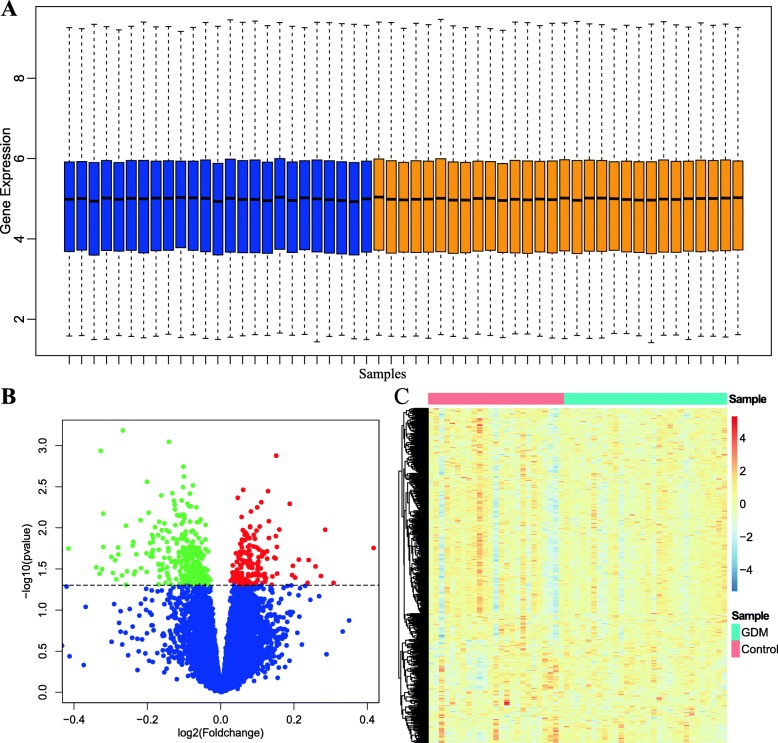
Identification of DEGs between GDM and healthy controls samples. (**a**) The box plot depicts the overall gene expression level of each sample after normalization (blue bars: normal sample, orange bar: GDM sample). (**b**) The volcano plot of DEGs. (**c**) The expression heatmap of DEGs

The gene microarray data of 55 samples were obtained from GEO database. After standardization and gene annotation, the expression profiles of 23,313 genes were obtained. The gene expression distribution of each sample was shown as Fig. 2a. A total of 465 DEGs were obtained between GDM and healthy samples, of which 165 genes were up-regulated in the healthy group, 300 genes were up-regulated in the GDM group. The volcano map was shown in Fig. 2b, and the expression heatmap of the DEGs was shown in Fig. 2c.

### Functional enrichment analysis of DEGs

**Fig. 3:**
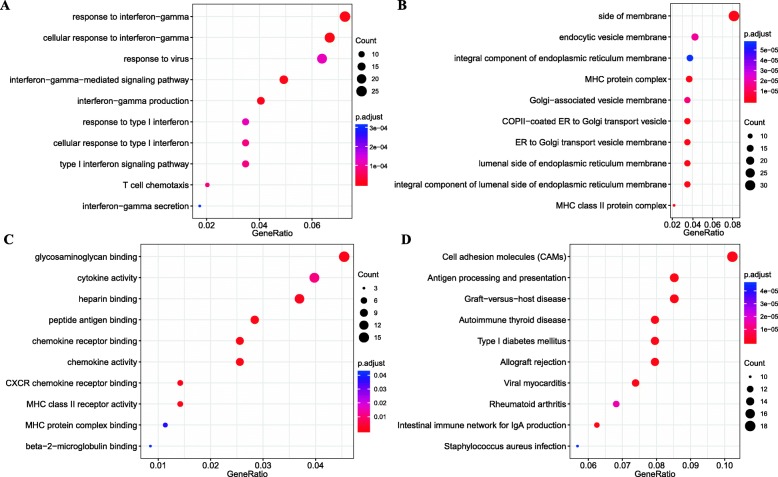
Functional enrichment analysis of 465 DEGs. (**a**) Enriched GO terms in the “biological process” category. (**b**) Enriched GO terms in the “cellular component” category. (**c**) Enriched GO terms in the “molecular function” category. (**d**) Enriched KEGG biological pathways. The x-axis represents the proportion of DEGs, and the y-axis represents different categories. The different colors indicate different properties, and the different sizes represent the number of DEGs

To better understand the functional implications of the 465 DEGs, GO and KEGG functional enrichment analysis was performed (Additional file 1: Table S1). In the biological process category, 108 enriched GO terms were observed. They were mainly enriched in response to interferon-gamma, T cell chemotaxis, and type I interferon signaling pathway (Fig. 3a). These results suggested a link between insulin resistance and the immune pathway. Insulin resistance was reported as the result of an inflammatory environment [31]. Categorization by “cellular component” revealed 41 enriched GO terms, and they were mainly associated with MHC protein complex and lumenal side of endoplasmic reticulum membrane (Fig. 3b). Moreover, the “molecular function” category revealed 14 significant enrichment in GO terms associated with the MHC class II receptor activity, and chemokine receptor binding (Fig. 3c).

The KEGG enrichment analysis revealed 30 biological pathways such as Type I diabetes mellitus, Cell adhesion molecules (CAMs), and Intestinal immune network for IgA production (Fig. 3d). It was worth mentioning that Type I diabetes mellitus was associated with GDM. In short, these DEGs were closely related to immunity, MHC, and diabetes mellitus.

### KEGG pathway gene interaction network

**Fig. 4:**
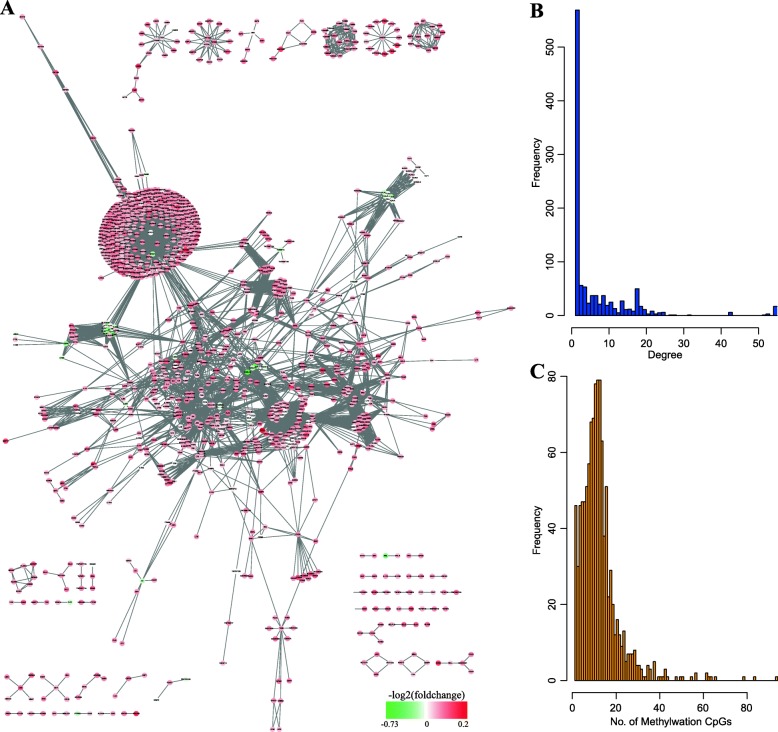
KEGG pathway gene interaction network analysis. (**a**) KEGG pathway gene interaction network. The colors indicated different fold-change. (**b**) The distribution of network degree. (**c**) The distribution of network methylation CpG sites in the promoter region

We then downloaded the XML file of 30 enriched pathways from the KEGG website, extracted the gene interaction information by the XML R package, and converted the gene id into gene symbol. Finally, we constructed a KEGG pathway gene interaction network, which had a total of 1091 genes with expression levels with 4169 interactions. As shown in Fig. 4a, most of them were down-regulated in GDM. In the further analysis of network topology properties, the network degree distribution was shown in Fig. 4b. We found that the proportion of nodes with large degree was small, and most node degrees were small and exhibited power law distribution, which was consistent with the distribution characteristics of biomolecular network. In the analysis of methylation sites of gene promoter regions in the network, we found that a total of 1013 (92.9%) genes with methylation sites at promoter region. The number of methylation sites was as shown in Fig. 4c, and there were 876 (82.5%) genes, whose promoter regions with methylated CpG sites were below 20.

### Identification of key epigenetics driven genes in GDM

**Fig. 5:**
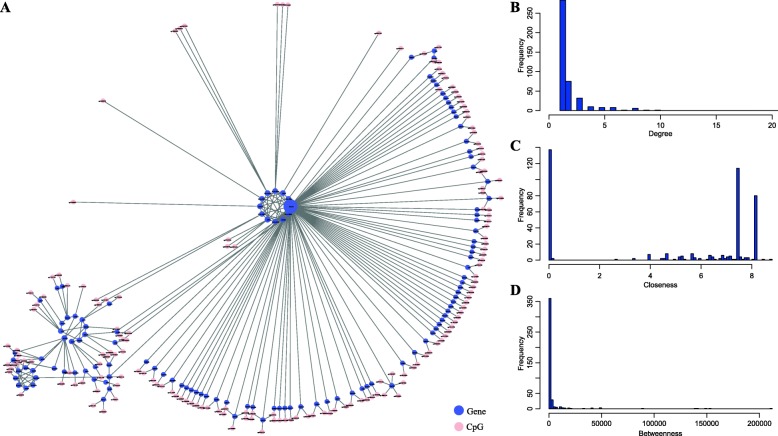
Identification of key epigenetics driven genes in GDM. (**a**) Gene-gene-CpG interaction network, in which the pink dot was methylated CpG, the blue dot represented the gene. (**b**) The degree distribution of the network. (**c**) The network Closeness distribution. (**d**) The network Betweenness distribution

We analyzed the correlations between KEGG pathway gene expression and promoter methylation sites. A total of 184 (18.2%) genes with 242 methylation sites were significantly negatively correlated with their promoter region methylation (Additional file 2: Table S2). These genes were potentially key epigenetics driven genes that were linked to promoter methylation sites to form a gene-gene-CpG interaction network (Fig. 5a). The degree distribution of the network was shown in Fig. 5b, and the power law distribution was also presented. Moreover, we calculated the Closeness of this network. It was found that most nodes had lower Closeness, and a few nodes had higher Closeness (Fig. 5c). The network Betweenness distribution was shown in Fig. 5d, and most nodes had low Betweenness; high degree, high Closeness or high Betweenness were considered to be important in the network. Next, we chose the node that satisfies the top 10% degree, Closeness, and Betweenness as 10 epigenetics driven hub genes (STAT1, HLA-DOA, HLA-DMB, HLA-DQB1, HLA-DRB1, HLA-DMA, HLA-DRA, HLA-DPB1, IFNGR2, EIF2AK2), wherein HLA-DMB, HLA-DMA, HLA-DQB1, HLA-DRB1, HLA-DRA, HLA-DPB1 were HLA class II histocompatibility antigen. The main genomic region controlling the predisposition to type 1 diabetes was the Human Leukocyte Antigens (HLA) class II of the major histocompatibility complex [32]. HLA-DRB1 was proved to increase insulin secretion and reduce the risk of type 2 diabetes [33]. STAT1 mutation was closely related to type 1 diabetes susceptibility [34]. EIF2AK2 was overexpressed in islets of type 1 diabetes patients [35]. In total, these hub genes were closely related to the development of diabetes, and these 10 genes may be used as GDM markers.

### Construction of diagnostic models and validation

**Fig. 6:**
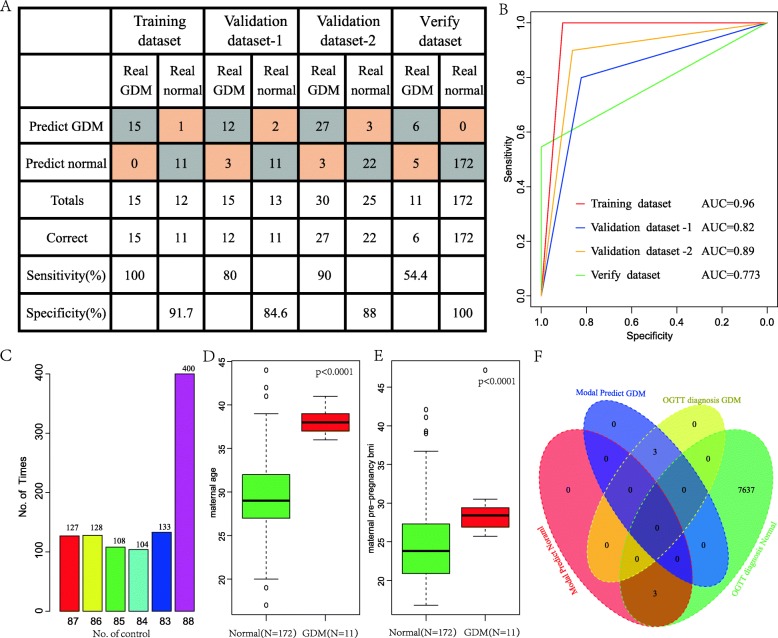
Construction of diagnostic models and validation. (**a**) The classification result of the diagnostic model in the training data set, verification data set and GSE128381 data set. (**b**) The ROC curve of diagnostic model in the training data set, verification data set and GSE128381 data set. (**c**) The number of normal samples predicted by the prediction model in a thousand random normal samples. (**d**) Age distribution difference of pre-pregnancy between GDM samples and normal samples, and t-test was used to calculate the p value. (**e**) BMI distribution difference of pre-pregnancy between GDM samples and normal samples, and t-test was used to calculate the *p* value. (**f**) Relationship between model prediction results and OGTT diagnostic results

We randomly divided 55 samples into two groups, one group as training data set (*n* = 27, GDM = 15, Normal = 12), and one group as validation data set (*n* = 28, GDM = 15, Normal = 13). In training dataset, 10 hub genes were used as features to obtain their corresponding expression profiles, and then the SVM classification model was constructed. The model test used a ten-fold cross-validation method with a classification accuracy of 96.3% (Fig. 6a) and 26 of 27 samples were classified correctly. The model has a sensitivity to GDM of 100% and a specificity of 91.7% with the AUC of 0.96 (Fig. 6b). Further, we used the established model to predict the samples in the validation data set to test the predictive power of this model. Twenty-three of 28 samples were correctly classified and the classification accuracy was 82.1%. The model had a sensitivity of 80% for GDM and a specificity of 84.6% (Fig. 6a). The AUC value was 0.82 (Fig. 6b). Finally, all samples were predicted using above established model to test the predictive power. Forty-nine of 55 samples were correctly classified, with a classification accuracy of 89.1%. The model had a sensitivity of 90% for GDM and a specificity of 88% (Fig. 6a). The AUC value was 0.89 (Fig. 6b). These results indicated that the diagnostic prediction model constructed in this study can effectively distinguish between GDM patients and normal controls. These 10 epigenetics driven genes may be used as reliable biomarkers for GDM diagnosis.

### The superiority of diagnostic model in the external verification set

A separate set of data sets GSE128381 was selected, containing 183 Placental tissue samples, and the model was applied to these pregnant Placental tissue samples to analyze the accuracy of the model. Specifically, we selected a set from the Hasselt University Centre for The Environmental Sciences data set, GEO number is GSE128381, the expression matrix of 10 hub genes were extracted, our model was used to predict the sample and compared it with the clinical diagnosis. Among them, 178 of the 183 patients diagnosed as normal samples were predicted as normal samples, and 5 of the 6 patients diagnosed as GDM were predicted as GDM patients, with an accuracy rate of 97.3% (Fig. 6a), the area under the ROC curve was 0.773 (Fig. 6b), and the overall prediction performance was good, and a good predictive performance across data platforms. Furthermore, 88 (50%) samples were randomly selected from 177 known normal women using our model for prediction, and the number of normal samples was statistically predicted. In order, 1000 times were randomly selected, among which 400 (40%) times were correctly classified 100%, 5 (5.6%) were the biggest prediction errors, and the frequency was 133 (13.3%) times (Fig. 6c). This indicated that the model has good stability. To analyze the relationship between the model and the maternal history, the 183 cases from Hasselt University Centre for Environmental Sciences were predicted to be GDM group and normal group. The characteristics of the two groups of pregnant women were analyzed, and we found the age of the pregnant women predicted to be GDM were significantly higher than that the predicted normal sample (Fig. 6d). The pre-pregnancy BMI comparison also showed that the GDM sample was significantly higher than normal (Fig. 6e). It is well known that age and BMI are risk factors for GDM in pregnant women, and the model is consistent with maternal age and BMI. To run the double-blind trial, we used the expression profiles of HUVEC cells from umbilical cords in six pregnant women tested by Ambra R et al. [30], our model was used to predict and identify three GDM and three normal samples. The oral glucose tolerance test (OGTT) was further performed between the 24th and 34th gestational weeks, and the three GDMs reported by the GTT were completely consistent with the model predictions. Furthermore, the expression profiles of Placental tissue samples from 183 pregnant women tested by Cox B et al. [29] were predicted by our model to identify 11 GDM samples and 172 healthy group samples, However, according to clinical diagnosis of Cox B et al., 5 of the 11 predicted GDM samples were diagnosed as GDM, and 172 predicted healthy samples were all diagnosed as normal samples (Fig. 6f). This suggests that the model is suitable for different data platforms and is highly consistent with current clinical diagnostic methods.

## Discussion

Some studies have showed that patients with GDM suffer a higher risk of developing type I/II diabetes in the future than normal pregnant women [36]. Type I/II diabetes will be a major healthy burden without proper medical intervention. In this study, we compared the differences in gene expression between GDM and healthy control samples. Function analysis of these DEGs revealed that these genes were mainly enriched in immune, histocompatibility complex (MHC) and type I diabetes-related pathways. The underlying pathogenesis of type I diabetes in GDM may be associated with autoimmunity. Type I diabetes was characterized by progressive destruction of pancreatic beta cells due to T cell-mediated autoimmunity, leading to insulin deficiency and hyperglycemia. Polymorphisms in the class II human leukocyte antigen (HLA) gene encoded by the MHC region were related to susceptibility in type 1 diabetes [37]. These class II molecules play important roles in antigen-peptide presentation-assisted T cells.

DNA methylation was an indispensable epigenetic modification which inhibited transcription of a gene by inhibiting the binding of specific transcription factors [38]. Hyperglycemia in the uterine environment may also induced epigenetic adaptation, led to DNA methylation changes, thus affected the risk of obesity and type 2 diabetes in future generations [39]. We combined the gene expression and gene promoter methylation to screen for genes those regulate abnormalities from the GDM-related KEGG pathway gene regulatory network, and further screened hub genes such as STAT1, HLA-DOA, and HLA-DMB, HLA-DQB1, HLA-DRB1, HLA-DMA, HLA-DRA, HLA-DPB1, IFNGR2, and EIF2AK2. The literature mining found that most of these genes were associated with type I diabetes.

In addition, pregnant women with gestational diabetes are prone to miscarriage early in pregnancy, and impaired fetal development may lead to glucose intolerance and obesity in infants [40]. Therefore, early diagnosis and personalized medical intervention of GDM are of great significance. Previously, Wang et al. [41] has established a diagnostic model by using six gene expression profiles, but the AUC was relatively low. In this study, the SVM was used which based on 10 hub genes for GDM. The gene expression profile was constructed and verified by a classifier. The AUC reached 0.96 in the training set, indicating that these genes have a good classification effect on GDM. The AUC in the validation data set also reached 0.82. Our double-blind trial that the model is suitable for different data platforms and is highly consistent with current clinical diagnostic methods. These results indicated that these 10 genes may be regard as GDM diagnostic markers, which provided targets and references for clinicians.

Although we identified potential candidate genes involved in GDM development in large samples through bioinformatics techniques, we should be aware of several limitations of this study. First, the samples lack for clinical follow-up information, so we did not consider other factors such as the presence of other health status of the patients to distinguish GDM diagnostic biomarkers. Second, it was inadequate that the results were obtained only by bioinformatics analysis, thus further experimental validation was needed to confirm above results, such as genetic analysis and experimental studies of larger sample sizes.

In summary, we systematically analyzed the methylation status of more than 20,000 gene expressions and 270,000 CpGs, and extracted key genes based on regulation relationships in GDM-related pathways. We found the expression characteristics of key genes, which were closely related to the development of type 1 diabetes in the GDM. Although our proposed gene expression profile still lacked the high specificity required for immediate diagnostic applications, GDM may be predicted with high accuracy (AUC = 0.96) from gene expression profiles in placental tissue for clinicians.

## Conclusions

In conclusions, this study provided new features for the diagnosis of GDM and may contributed to the diagnosis and personalized treatment of GDM.

## Supplementary information

**Additional file 1: Table S1.** The results of GO terms and KEGG pathway enrichment of DEGs

**Additional file 2: Table S2.** Identification of key epigenetics driven genes in GDM. A total of 242 methylation sites were significantly negatively correlated with their promoter region methylation

## Data Availability

The data used for supporting the results of the study are included within the article.

## Abbreviations

CAMs: Cell adhesion molecules
DEGs: differentially expressed genes
GDM: Gestational diabetes mellitus
GEO: Gene Expression Omnibus
GO: Gene Ontology
HLA: Human leukocyte antigen
IADPSG: International Association of Diabetes and Pregnancy Research Groups
KEGG: Kyoto Encyclopedia of Genes and Genomes
SVM: support vector machine
